# Chemically Mediated Interactions with Macroalgae Negatively Affect Coral Health but Induce Limited Changes in Coral Microbiomes

**DOI:** 10.3390/microorganisms11092261

**Published:** 2023-09-09

**Authors:** Jenny Fong, Peggy P. Y. Tang, Lindsey K. Deignan, Jovena C. L. Seah, Diane McDougald, Scott A. Rice, Peter A. Todd

**Affiliations:** 1 Experimental Marine Ecology Laboratory, National University of Singapore, Singapore 117558, Singapore; jovenaseah@u.nus.edu (J.C.L.S.); dbspat@nus.edu.sg (P.A.T.); 2 Singapore Centre for Environmental Life Sciences Engineering, Nanyang Technological University, Singapore 637551, Singapore; peiyipeg001@e.ntu.edu.sg (P.P.Y.T.); ldeignan@ntu.edu.sg (L.K.D.); diane.mcdougald@uts.edu.au (D.M.); scott.rice@csiro.au (S.A.R.); 3 Australian Institute for Microbiology & Infection, University of Technology Sydney, Sydney, NSW 2007, Australia

**Keywords:** allelopathy, competition, coral–macroalgal interaction, coral reef, Singapore

## Abstract

Allelopathic chemicals facilitated by the direct contact of macroalgae with corals are potentially an important mechanism mediating coral–macroalgal interactions, but only a few studies have explored their impacts on coral health and microbiomes and the coral’s ability to recover. We conducted a field experiment on an equatorial urbanized reef to assess the allelopathic effects of four macroalgal species (*Bryopsis* sp., *Endosiphonia horrida*, *Hypnea pannosa* and *Lobophora challengeriae*) on the health and microbiomes of three coral species (*Merulina ampliata*, *Montipora stellata* and *Pocillopora acuta*). Following 24 h of exposure, crude extracts of all four macroalgal species caused significant coral tissue bleaching and reduction in effective quantum yield. The corals were able to recover within 72 h of the removal of extracts, except those that were exposed to *L. challengeriae*. While some macroalgal extracts caused an increase in the alpha diversity of coral microbiomes, there were no significant differences in the composition and variability of coral microbiomes between controls and macroalgal extracts at each sampling time point. Nevertheless, DESeq2 differential abundance analyses showed species-specific responses of coral microbiomes. Overall, our findings provide insights on the limited effect of chemically mediated interactions with macroalgae on coral microbiomes and the capacity of corals to recover quickly from the macroalgal chemicals.

## 1. Introduction

Coral reefs are experiencing degradation globally due to the cumulative impacts of multiple local anthropogenic stressors (e.g., overfishing, eutrophication and disease) compounded by rising seawater temperatures and ocean acidification [1,2,3]. The declines in live coral cover are often accompanied by the proliferation of macroalgae [4,5], resulting in an increase in the competition between corals and macroalgae for space. Established macroalgal communities may generate positive feedback loops, which further reinforce macroalgal dominance and coral-depauperate state by promoting greater macroalgal recruitment [6], suppressing herbivory [7], reducing coral growth and survivorship [8,9], as well as inhibiting coral fecundity and recruitment [10,11]. Understanding the processes that mediate coral–macroalgal interactions is critical to the management and conservation of coral reef ecosystems [12].

The coral microbiome consists of dinoflagellates, bacteria, archaea, fungi and viruses [13]. Together, they play important roles in nutrient cycling, coral host immunity against pathogens and mediating environmental stress [13,14,15]. Macroalgae can negatively affect corals and their microbiomes through a range of physical, chemical and microbial mechanisms [16,17,18]. In addition to space pre-emption, macroalgae may physically damage corals via shading, abrasion and increased localized sedimentation rates [9,19]. Several studies have documented that some macroalgae possess allelochemicals, which can cause coral tissue bleaching and mortality [17,20] and reduce coral larval survivorship and settlement rates [21,22]. Some macroalgal chemicals have also been shown to possess antibacterial properties that differentially target marine bacteria, resulting in shifts to higher abundances of harmful microbes and/or lower abundances of beneficial microbes [21,23]. In addition, macroalgae may destabilize the coral microbiome by transmitting pathogenic microbes to corals upon contact [24] and by releasing a fraction of their photosynthetically fixed carbon as dissolved organic matter (DOM) into the water column [25]. Macroalgal DOM is readily consumed by microbes [26], which can lead to an overactivity in the coral microbiome, resulting in hypoxic microenvironments that may favor copiotrophic and potentially pathogenic microbes [25,27].

Previous studies have revealed that chemically mediated interactions between corals and macroalgae are dependent on direct contact [17,20,28]. Hydrophobic, lipid-soluble chemicals extracted from surfaces of macroalgae were embedded into gels and placed in contact with corals, causing coral bleaching and reduction in coral photosynthetic efficiency in 79% of the 24 coral–macroalgal interaction pairs assayed [17]. Similarly, extracts from eight *Lobophora* spp. Induced tissue bleaching in *Acropora muricata* and *Stylophora pistillata*, but not in *Porites cylindrica* and *Montipora hirsuta* [20]. The low solubility of lipid-soluble macroalgal extracts in water also suggests that these chemicals are most efficient when they are transferred to corals by direct contact rather than dissolution through water [17,28]. However, interactions between corals and macroalgae are complex, with varying allelopathic potential among macroalgal species and differential susceptibility among coral species [16,17,20]. Furthermore, it remains unclear how corals and their microbiomes recover from the damage caused by macroalgal chemicals.

To narrow this knowledge gap, a field experiment on an equatorial urbanized reef was conducted to assess the allelopathic effects of macroalgae on coral health and the prokaryotic portion of their microbiomes. Specifically, the effects of lipid-soluble extracts of four macroalgal species (*Bryopsis* sp., *Endosiphonia horrida*, *Hypnea pannosa* and *Lobophora challengeriae*) were tested on three coral species (*Merulina ampliata*, *Montipora stellata* and *Pocillopora acuta*). The responses of corals after the macroalgal extracts were removed were also examined to assess the coral recovery potential.

## 2. Materials and Methods

### 2.1. Macroalgal Crude Extracts

The allelopathic potency of lipid-soluble extracts from four common macroalgal species in Singapore were examined, namely the green alga *Bryopsis* sp., the brown alga *Lobophora challengeriae* and the red algae *Endosiphonia horrida* and *Hypnea pannosa*. These macroalgal species were selected because they interacted frequently with corals on Singapore’s reefs [29], and their chemical effects on adult corals and their microbiomes have not been explored previously. Samples were collected from three shallow fringing reefs in Singapore at depths of 2–3 m (Figure S1) and cleaned of epiphytes. Hydrophobic, lipid-soluble metabolites were extracted from the macroalgal surfaces, including their surface-associated microbiome, following the hexane dip technique [30]. Samples were first spun in a salad spinner to remove excess water and thereafter were extracted in 100% hexane for 30 s while being vortexed vigorously [30,31]. Extracts were dried in vacuo and stored at −20 °C. To prepare the extract gels, extracts were resuspended in 0.5 mL methanol and added to autoclaved molten Phytagel medium (Sigma–Aldrich, St. Louis, MI, USA) at natural volumetric concentrations (e.g., 10 mL volume of gel was prepared using 10 mL displacement volume of algae). The extract gel was poured in a mold fitted with plastic screen net, allowed to cool down and cut into 2 cm × 2 cm squares. Control gels were made in the same manner using only methanol without the addition of macroalgal extracts. Gels were stored at −20 °C for two weeks until deployment in the experiment.

### 2.2. Experimental Procedures

The effects of algal extracts were tested on the colonies of *Merulina ampliata*, *Montipora stellata* and *Pocillopora acuta* growing at depths of 2–3 m on Pulau Satumu, Singapore (Figure S1). These corals were selected as they are common on Singapore’s reefs [32] and within genera that have been shown to vary in susceptibility to macroalgal competition [33]. They also have branching morphologies, which made it easier to attach the gels. Fifteen colonies of each coral species (>15 cm diameter), which had no signs of bleaching, were tagged. On each colony, five extract gels (one control and each of the four macroalgal species) were secured to different coral branches using cable ties (following Ref. [28]). While a blocked experimental design may result in algal treatments having synergistic effects on coral colonies, previous studies have demonstrated that the effects of macroalgae and their extracts were localized to the area of direct contact [17,28,31]. The use of a blocked design also minimized the effect of inter-colony variation, especially in coral microbiomes, among algal treatments. Finally, the design substantially reduced the number of coral colonies that had to be sacrificed—from 75 colonies per coral species when using a complete independent design to 15 colonies per coral species when using a blocked design.

After 24 h, the gels were removed from all colonies, and the impacts of macroalgal extracts on coral health were assessed. The coral tissue underneath the area occupied by each gel was photographed to quantify the percentage of coral tissue bleached using ImageJ v1.52n [34] (Figure S2). The effective quantum yield of each coral branch where gels were applied was also measured using a portable diving pulse amplitude modulated (PAM) fluorometer (Heinz Walz GmbH, Effeltrich, Germany). Cable ties, which were used to secure the extract gels, were left at the base of the coral branches to mark the location of the coral branches where treatments were applied.

To assess the recovery potential of corals, the percentage of coral tissue bleached and effective quantum yield were measured 24 h and 72 h after gels were removed. At each time point (i.e., 24 h after gels were applied, ‘day 0’; 24 h after gels were removed, ‘day 1’; and 72 h after gels were removed, ‘day 3’), the coral branches that were exposed to macroalgal treatments were also collected from five colonies of each coral species for microbial analysis (hence, the number of available replicates for the percentage of coral tissue bleached and effective quantum yield was reduced by five at each time point). All PAM measurements were taken between 11:30 and 14:00. Coral fragments were put in individual sterile sampling bags and transported on ice to the laboratory. Samples were stored in a −80 °C freezer until further processing.

### 2.3. Coral Microbiome Analysis

Tissues from the coral fragments were extracted using the WaterPik method. DNA extraction was carried out using the Qiagen DNeasy PowerBiofilm Kit and Zymo Clean & Concentrator-25 kit, resulting in 30 µL of eluted DNA for each coral tissue sample. PCR was carried out to amplify the 16S rRNA V4 region with the 515F-806R primer set [35,36,37]. The PCR was run as follows: 1 cycle of 95 °C for 5 min, 35 cycles of 94 °C for 30 s, 35 cycles of 53 °C for 40 s, 35 cycles of 72 °C for 1 min, 1 cycle of 72 °C for 10 min, followed by rest at 4 °C. Each PCR reaction mix consisted of 10 µL of Qiagen HotStar Taq Plus Master Mix, 1 µL of 10 µM 515F and 806R primers each, 1 µL of 400 µM Bovine Serum Albumin, 5 µL Ambion nuclease-free water (Carlsbad, CA, USA) and 2 µL of 5 ng µL^−1^ template DNA.

For *M. ampliata* and *M. stellata*, PCR products were subjected to magnetic bead clean-up with AMPure magnetic beads and were eluted in 50 µL TE buffer. For *P. acuta*, PCR products were run on 2% agarose gel, excised with the Invitrogen PureLink Quick Gel Extraction Kit and eluted in 50 µL kit Elution Buffer (TE buffer). All PCR products were quantified with Invitrogen Qubit DNA High Sensitivity Kit, followed by visualization using the BioAnalyzer with Agilent D1000 Screentapes. The purified PCR products were submitted to the sequencing facility at the Singapore Centre for Life Sciences Engineering for amplicon sequencing using the Illumina MiSeq300 platform.

### 2.4. Statistical Analyses

To evaluate the allelopathic effects of macroalgal crude extracts on corals, linear mixed-effect (LME) models were performed using the *lme* function from the *nlme* package [38] in R v4.2.1 [39]. The percentage of tissue bleaching and effective quantum yield of corals were fitted as response variables, while macroalgal treatment, coral species and their interactions were fitted as fixed factors. Colony identity was included as a random effect to account for the blocked experimental design. Separate analyses were conducted for each time point due to unequal number of samples (i.e., *n* = 15 for day 0, *n* = 10 for day 1 and *n* = 5 for day 3). Significance tests of fixed factors were determined based on likelihood ratio tests and changes in Akaike’s information criterion (AIC). Post hoc Dunnett’s tests for comparison of each treatment against control were conducted using the *emmeans* package [40]. Where necessary, the residual variance was allowed to differ among coral species and macroalgal treatment to control for heteroscedasticity. Assumptions of normality and homoscedasticity were validated by visual inspection of the models’ residual plots.

Coral microbial data were processed following DADA2 pipeline v. 1.16 [41] to generate amplicon sequence variants (ASVs). In summary, the sequences were trimmed with the *trimLeft* and *truncLen* parameters set to (52, 52) and (190, 190), respectively, followed by dereplication, merging of the paired reads and removal of chimeric sequences. Taxonomic data were assigned to the sequences using *dada2*-formatted reference files developed from the Silva-ARB version 138.1 database with the *assignTaxonomy* and *addSpecies* functions. Subsequent analyses of coral microbiome data were performed separately for each coral species, as they had distinct microbial communities (PERMANOVA: *F* = 38.7, *p* = 0.001; Figure S3).

Three alpha diversity indices (chao1, Shannon–Wiener, inverse Simpson) were generated using *phyloseq* [42]. LME models were fitted to determine whether the alpha diversity indices were significantly different among macroalgal treatments and time points. Non-metric multi-dimensional scaling (NMDS) plots using Bray–Curtis dissimilarity of square-root-transformed data were generated to examine the patterns in the beta diversity of coral microbiomes. Permutational multivariate analysis of variance (PERMANOVA) and permutational analysis of multivariate dispersions (PERMDISP) were carried out to compare the differences in the average community composition of coral microbiomes and their variability among macroalgal treatments and time points. The *DESeq2* package [43] was also used to determine which ASVs had significant difference in abundance when the microbiomes between the control and treated microbiomes were compared for each experimental time point.

## 3. Results

**Figure 1:**
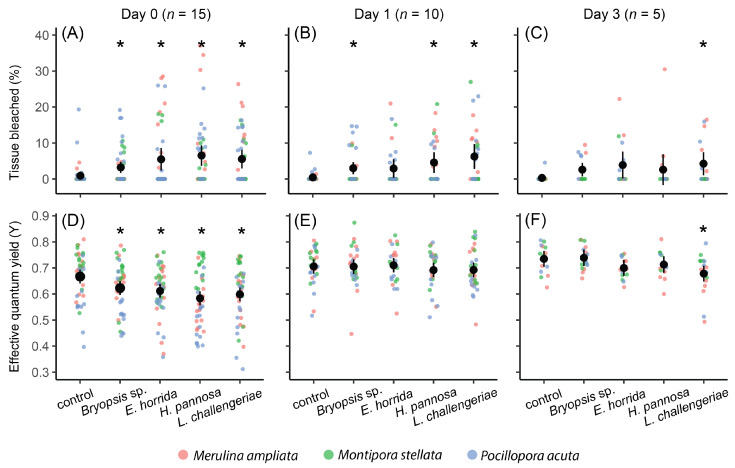
Effects of macroalgal crude extracts (estimated marginal means and 95% confidence intervals) on the percentage of coral tissue bleached (**A**–**C**) and effective quantum yield (**D**–**F**). Asterisks indicate significant differences between control and macroalgal gel treatments for all three coral species based on Dunnett’s post hoc comparisons.

Coral tissue health and effective quantum yield differed significantly among the macroalgal gel treatments (Table 1, Figure 1). At day 0, compared to the control gels, coral branches that were exposed to the crude extracts of *Bryopsis* sp., *E. horrida*, *H. pannosa* and *L. challengeriae* had a significantly higher percentage of tissue bleached and lower effective quantum yield (Figure 1). Twenty-four hours after the extracts were removed (i.e., day 1), corals that were previously exposed to *Bryopsis* sp., *H. pannosa* and *L. challengeriae* extract gels had significant tissue bleaching (Table 1, Figure 1). In contrast, 72 h after the extracts were removed (i.e., day 3), only corals that were treated with *L. challengeriae* extract gels had significant tissue bleaching and lower effective quantum yield compared to the control gels (Table 1, Figure 1). While the percentage of tissue bleached and effective quantum yield differed significantly among corals, there were no significant interactions between coral and macroalgal treatments (Table 1).

**Figure 2:**
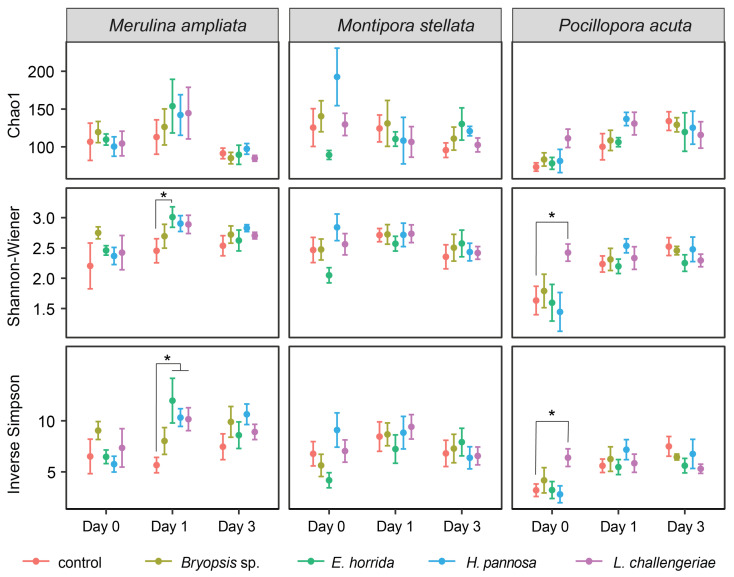
Variations in the alpha diversity indices of the coral microbiomes (mean ± SE) when in contact with various macroalgal extracts sampled at different time points. The colors indicate the type of macroalgal extract, while the asterisks indicate significant differences compared to the control.

Coral microbiomes were dominated by members of the Phyla Proteobacteria, Bacteroidota and Firmicutes, while other Phyla, such as Bdellovibrionota, Cyanobacteria, Planctomycetota and Verrucomicrobiota, were present in lower abundances (Figure S4). Macroalgal extracts caused significant changes in the alpha diversity indices of coral microbiomes, but the changes were variable among coral species (Figure 2). For *M. ampliata*, significant differences in alpha diversity indices were observed on day 1: the *E. horrida* treatment had higher Shannon–Wiener and inverse Simpson indices than the controls, while *H. pannosa* and *L. challengeriae* treatments had a higher inverse Simpson index than the controls (Figure 2). For *P. acuta*, significantly higher Shannon–Wiener and inverse Simpson indices were observed in corals exposed to *L. challengeriae* extracts compared to controls on day 0. In contrast, the diversity of *M. stellata* microbiomes remained relatively stable among treatments at each time point (Figure 2).

**Figure 3:**
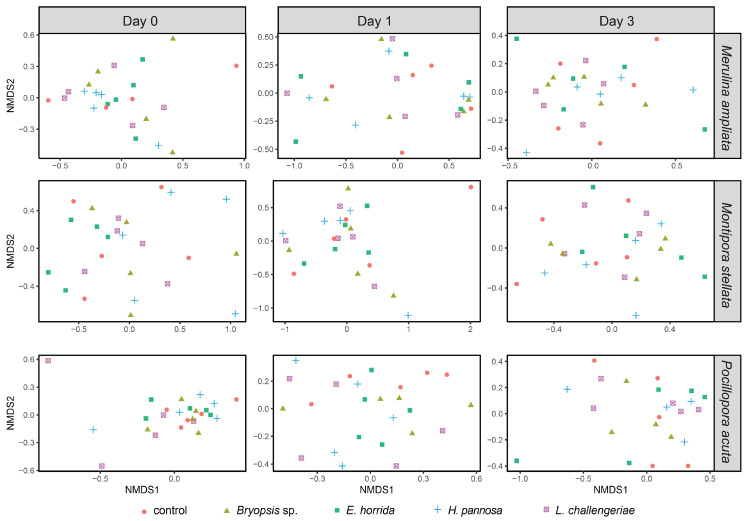
Non-metric multi-dimensional scaling (NMDS) plots showing the community composition of coral microbiomes at each sampling time point for *M. ampliata*, *M. stellata* and *P. acuta*. The stress values of the nMDS plots were <0.2.

There were shifts in the coral microbial community compositions across time points, including the control gels (Figure S5). Therefore, to assess the specific response of the coral microbiomes to macroalgal treatments, we focused on the comparisons between coral fragments that received algal extracts and control gels at each time point. PERMANOVA and PERMDISP showed that on day 0, the coral microbiomes did not vary significantly among macroalgal treatments following 24 h of extract exposure (Table 2, Figure 3). During the recovery period (day 1 and 3), there were also no significant differences in the average composition and variability of coral microbial communities between control gels and macroalgal extracts (Table 2, Figure 3). However, DESeq2 analysis revealed that there were many ASVs that had significant changes in abundance (Table 2). In *M. stellata*, the greatest ASV changes were observed on day 0, while the highest number of ASV changes in *M. ampliata* and *P. acuta* occurred on day 1. Notably, among the three coral species, *M. stellata* had the greatest number of ASVs that differed in abundance across the time points. The ASVs were mostly members of the Phyla Proteobacteria, Firmicutes, Bacteroidota, Actinobacteriota, Bdellovibrionota and Verrucomicrobiota (Table S1).

## 4. Discussion

Coral–macroalgal interactions are common on coral reefs; yet, the mechanisms mediating them remain poorly understood. In this study, we found that lipid-soluble extracts of *Bryopsis* sp., *E. horrida*, *H. pannosa* and *L. challengeriae* caused significant tissue bleaching and reduction in effective quantum yield across the three coral species tested. However, corals were able to recover within 72 h of the removal of macroalgal extracts, except those that were exposed to *L. challengeriae*. The effects of the macroalgal extracts on the coral microbiomes were less pronounced. Some extracts caused an increase in the microbial alpha diversity, but there were no significant differences in the average composition and variability of coral microbiomes among macroalgal treatments. However, marked changes in the abundance of individual ASVs were observed, particularly in *M. ampliata* microbiomes. Overall, our findings suggest that chemically mediated interactions with macroalgae have limited impacts on coral microbiomes at the time scales we tested, and corals are able to recover quickly from the damage by macroalgal chemicals.

Hydrophobic surface extracts from all four macroalgal species caused significant tissue bleaching and decline in effective quantum yield. Extracts from *E. horrida*, *H. pannosa* and *L. challengeriae* also caused an increase in the alpha diversity of coral microbiomes. While we did not identify and isolate the active allelopathic compounds responsible for affecting the corals, our results corroborate findings from past research, which demonstrated that these macroalgae—potentially with their surface-associated microbiomes—contained allelochemicals. An active anti-predatory depsipeptide compound known as kahalalide F was found in *Bryopsis* sp. from Hawaii [44]. Three halogenated terpenoids isolated from *H. pannosa* have been shown to possess antimicrobial properties [45,46]. The genus *Lobophora* is known to be chemically rich [20,47,48]. Three polyunsaturated alcohols from *L. rosacea* were identified to cause significant tissue bleaching in *Acropora muricata* [20]. Similarly, a compound isolated from *L. variegata* known as SQDG was responsible for inducing tissue bleaching in *Montastraea cavernosa* [48]. Aqueous crude extracts of *Lobophora* sp. were also found to exhibit broad-spectrum antibacterial activity and caused significant changes in coral microbiomes [21,49]. The results from the present study were largely consistent with previous bioassays, which demonstrated that *Bryopsis* sp., *Endosiphonia horrida* and *Lobophora* sp. crude extracts were chemically potent [22]. However, while *H. pannosa* extracts did not exert any effect on coral larvae [22], we found that they negatively affected coral health and increased the inverse Simpson index in *M. ampliata* microbiomes. These differences were likely due to the different extraction techniques used. The crude extracts of *H. pannosa* in Ref. [22] were obtained through whole-cell extraction; therefore, the extracts likely contained a range of both non-polar and polar compounds originating from both internal and external macroalgal cells. In contrast, the current study employed the hexane dip extraction technique to obtain lipid-soluble chemicals present on the surfaces of the macroalgae because chemical interactions between macroalgae and adult corals are most frequently mediated through direct contact [17]. It is also possible that the allelochemicals of *H. pannosa* act primarily on coral adults instead of coral larvae.

We found that the impacts of hydrophobic, lipid-soluble macroalgal extracts on coral microbiomes were limited. While some measures of alpha diversity of *M. ampliata* and *P. acuta* microbiomes were higher following exposure to the extracts compared to control gels, the average composition and variability of coral microbiomes were not altered by contact with them. While multiple studies have documented the shifts in coral microbiomes when corals were in contact with live macroalgae [49,50,51,52] but see [53], the impacts of macroalgal extracts on coral microbiomes were variable depending on their polarity and the species tested [21,23]. Aqueous, hydrophilic extracts of *Lobophora* sp. in the Caribbean altered the microbiomes of *Porites astreoides* and *Orbicella faveolata*, while the non-polar, lipophilic extracts of *Lobophora* sp. had contrasting effects on the coral microbiomes depending on the site and coral species tested [23]. Similar to our findings, macroalgal extracts were also found to damage coral tissue without altering coral microbial communities and vice versa [23]. Both hydrophilic and lipophilic crude extracts of *Lobophora* sp. in Australia were found to inhibit growth of bacterial isolates, but only hydrophilic extracts of *Lobophora* sp. induced shifts to *Vibrio* sp. dominance in *Porites cylindrica* microbiomes [21]. In this study, we only tested lipid-soluble crude extracts because for allelopathy to be effective, defensive chemicals are usually hydrophobic and transferred by contact [28,31]. Our findings add to a growing body of evidence that lipid-soluble extracts of macroalgae have limited impacts on coral microbiomes, suggesting that changes in coral microbiomes when in contact with live macroalgae are likely mediated by the transmission of macroalgal pathogens upon contact [24] or from the increase in the availability of DOM released by macroalgae [25,26]. It should be noted that our findings do not rule out the potential for macroalgae to alter coral microbiomes via polar chemicals mediated by indirect, water-mediated interactions, but further studies will be needed to determine the extent to which the polar fractions of macroalgal allelochemicals can influence coral microbiomes in field conditions.

Our results, however, did reveal a number of microbial ASVs with significant differences in abundance among the macroalgal treatments. These differences were influenced strongly by coral species. In *M. stellata* microbiomes, the number of differing ASVs ranged from 39 to 61 across the experimental sampling time points, with many ASVs in *M. stellata* microbiomes (22–37 ASVs) consistently having lower abundance following exposure to the macroalgal extracts. In contrast, there were between 3 and 39 ASVs that showed significant changes in abundance in *M. ampliata* and *P. acuta* microbiomes, with the largest changes observed at day 1. Certain ASVs, such as those associated with Rhodobacteraceae (e.g., ASV66) and *Alteromonas* sp. (e.g., ASV276), increased in abundance in *M. stellata* and *P. acuta* microbiomes that were treated with *Bryopsis* sp. and *L. challengeriae* extracts. Although these bacterial groups have been associated with the coral core microbiome [54], they have the potential to become opportunistic pathogens when conditions promoting dysbiosis occur [55,56]. Species-specific increases in ASVs associated with functions beneficial to coral health were also found 24 h after the removal of algal extracts, including ASVs associated with antimicrobial activity (e.g., *Erythrobacter* sp. (ASV47) and *Paracoccus* sp. (ASV69)) in *M. ampliata* and *M. stellata* microbiomes [57]. The relative abundance of *Peredibacter* sp. (ASVs 146, 285), which belongs to a group of predatory bacteria [58,59], was also higher in *P. acuta* microbiomes that were treated with *Bryopsis* sp. and *E. horrida* extracts, which likely served as a form of pathogenic bacterial control. On day 3 (i.e., 72 h after the removal of the extracts), the abundance of the ASVs associated with known pathogenic microbial groups, including Vibrionaceae (ASV157) and *Sphingomonas* sp. (ASV244) [60], had decreased across the macroalgal-treated microbiomes compared to control microbiomes in all three coral species. These changes in the abundances of ASVs suggest that the coral microbiomes were actively involved in maintaining and restoring coral health following sub-lethal stress caused by macroalgae. Future studies should concurrently assess coral holobiont transcriptome and microbiome responses to better understand how coral-associated microbes may influence the coral holobiont’s response to the competitive interactions with macroalgae.

Our findings indicate that corals can—within the time frame of exposure in the current study—mostly recover from the allelopathic impacts of macroalgae within a short period of time. *Merulina ampliata*, *M. stellata* and *P. acuta* recovered within 24 h of exposure to *Bryopsis* sp. and *E. horrida* extracts and within 72 h of exposure to *H. pannosa* extracts. Only corals that were exposed to *L. challengeriae* extracts did not recover within 72 h of the extracts being removed, suggesting that *L. challengeriae* is the most chemically potent among the macroalgal extracts tested. A previous study showed that six months after the removal of *Sargassum*, the growth rates of *Acropora millepora* and *Porites cylindrica* fragments that were previously surrounded by *Sargassum* were comparable to the growth rates of control fragments [61]. Another study reported that tissue damage on *Porites lobata* colonies caused by contact with *Galaxuara filamentosa* recovered within two weeks of the macroalgae being removed [33]. Our results, together with those from Refs. [33,61], demonstrate that corals are able to recover from macroalgal impacts. However, this might be dependent on whether the interaction frequency between corals and macroalgae can be limited temporally, e.g., by herbivory and macroalgal seasonality.

Previous studies have documented among-species variation in coral susceptibility to macroalgal allelopathic effects [17,33]. For instance, *Pocillopora damicornis* was reported to be more susceptible to macroalgal allelopathic damage than *Montipora digitata* [17,33]. Extracts of *Halimeda tuna* caused shifts in the bacterial communities of *Porites astreoides* but not *Orbicella faveolata*, while extracts of *Lobophora* sp. induced shifts in both corals [23]. In contrast, we did not find significant interaction effects of coral species and macroalgal treatments on the percentage of coral tissue bleached and effective quantum yield, implying that *M. ampliata*, *M. stellata* and *P. acuta* were equally susceptible to chemical damage by macroalgae. Further, the limited impacts of macroalgal extracts on the overall coral microbial compositions were consistent among the three coral species. These results align with a previous study, which documented the physiology and microbiomes of *M. ampliata*, *M. stellata* and *P. acuta* being similarly compromised by direct contact with *Lobophora* sp. and *H. pannosa* [51]. Our findings and those of Ref. [51] provide strong evidence that *M. ampliata*, *M. stellata* and *P. acuta* on Singapore’s reefs are equally susceptible to macroalgal competition.

In summary, our experiment showed that lipid-soluble chemicals found on surfaces of common macroalgae in Singapore can damage a range of coral species. However, the impacts of these chemicals on coral microbiomes were limited to changes in abundances of individual ASVs but not the overall microbial community compositions. Most corals recovered within short periods of time after the chemicals were removed, suggesting resistance to macroalgal damage. Determining the ability of corals to recover from the impacts of macroalgae is critical to understanding reef resilience and potential reversal of degraded reefs to coral-dominated reefs. Management strategies controlling macroalgal proliferation, such as herbivore protection or active macroalgal removal, may be necessary to facilitate coral recovery on reefs that are becoming algal dominated.

## Figures and Tables

**Table 1:** Summary of LME models on the effects of macroalgal treatment and coral species on the percentage of tissue bleached and effective quantum yield.

Variables	% Tissue Bleached		Effective Quantum Yield	
	∆AIC	*p*-Value	∆AIC	*p*-Value
Day 0				
Coral	−6.86	0.004	−30.7	<0.001
Macroalgae	−28.8	<0.001	−13.3	<0.001
Coral:Macroalgae	3.96	0.150	1.53	0.070
Day 1				
Coral	−9.25	0.001	−6.07	0.007
Macroalgae	−23.4	<0.001	6.53	0.831
Coral:Macroalgae	8.11	0.444	14.2	0.987
Day 3				
Coral	0.81	0.203	−2.43	0.040
Macroalgae	−9.02	0.002	−6.01	0.007
Coral:Macroalgae	3.51	0.131	10.0	0.652

**Table 2:** Summary of the PERMANOVA, PERMDISP and DESeq2 results of the coral microbiome composition and changes in the number of ASVs between the control and treated samples for each experimental time point. Details of the ASV changes can be found in Table S1.

Coral	Time Point	PERMANOVA		PERMDISP		DESeq2	
		F-Test	*p*-Value	F-Test	*p*-Value	Increase	Decrease
*M. ampliata*	Day 0	0.662	0.900	1.280	0.301	2	1
	Day 1	0.580	0.056	0.165	0.947	5	16
	Day 3	0.545	0.904	0.769	0.551	7	7
*M. stellata*	Day 0	0.916	0.057	0.597	0.662	39	22
	Day 1	0.523	0.381	0.254	0.897	10	29
	Day 3	0.569	0.617	0.425	0.799	11	37
*P. acuta*	Day 0	1.236	0.092	1.050	0.409	0	3
	Day 1	0.921	0.449	0.806	0.560	23	16
	Day 3	0.932	0.254	0.100	0.980	1	4

## Data Availability

The raw sequence data were uploaded to the NCBI Sequence Read Archive under the Bioproject ID PRJNA909298.

## Supplementary Materials

The following supporting information can be downloaded at: https://www.mdpi.com/article/10.3390/microorganisms11092261/s1, Figure S1: Map of the Southern Islands of Singapore showing the collection sites for macroalgae (*E. horrida* collected from Pulau Hantu, *L. challengeriae* and *Bryopsis* sp. from Pulau Subar Darat, and *H. pannosa* from Kusu Island) and Pulau Satumu, where the *in situ* experiment was conducted (A). Gray shaded areas represent fringing reef areas. Photos of macroalgal species (B–E) and coral species (F–H) tested in this study; Figure S2: Photos of coral branches (left: *Merulina ampliata*; right: *Pocillopora acuta*) suffering from tissue bleaching due to exposure to macroalgal extracts (left: *Lobophora challengeriae*; right: *Hypnea pannosa*); Figure S3: Non-metric multi-dimensional scaling (NMDS) plots based on Bray–Curtis distances comparing the coral microbiomes of the three coral species. The different colors indicate the three coral species used in this study; Figure S4: Relative abundances of the coral microbiomes (Phylum-level) of the three coral species that were in contact with macroalgal extracts across different time points; Figure S5: Non-metric multi-dimensional scaling (NMDS) plots showing the overall temporal shifts in the microbiomes of *M. ampliata*, *M. stellata* and *P. acuta*; Table S1: Summary of the *DESeq2* analyses showing the ASV differences in *M. ampliata*, *M. stellata* and *P. acuta* microbiomes between the methanol control and macroalgal extracts at different time points.
