# Supplementary material for: Chemically Mediated Interactions with Macroalgae Negatively Affect Coral Health but Induce Limited Changes in Coral Microbiomes
Source: Microorganisms. 2023 Sep 9;11(9):2261. doi: 10.3390/microorganisms11092261 (PMC10535309; doi:10.3390/microorganisms11092261)
Supplement: Supplementary file 1 [file microorganisms-11-02261-s001.zip › microorganisms-2593228-supplementary.pdf]

Chemically mediated interactions with macroalgae negatively affect coral health but induce limited changes in coral microbiomes.

Jenny Fong, Peggy P. Y. Tang, Lindsey K. Deignan, Jovena C. L. Seah, Diane McDougald, Scott A. Rice, Peter A. Todd

Supplementary Material

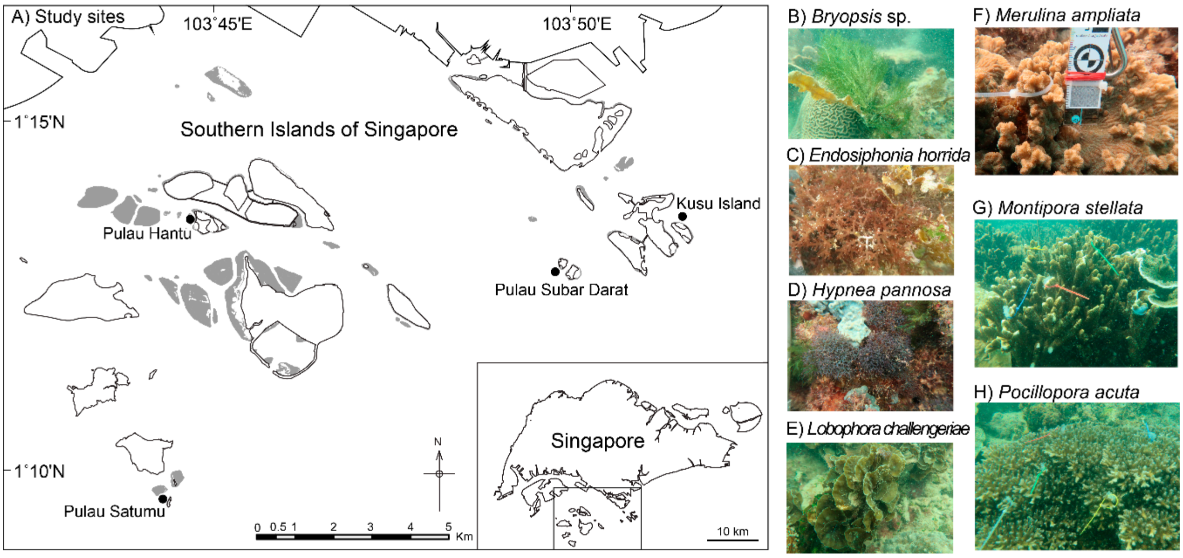

Figure S1. Map of the Southern Islands of Singapore showing the collection sites for macroalgae (*Endosiphonia horrida* collected from Pulau Hantu, *Lobophora challengeriae* and *Bryopsis* sp. from Pulau Subar Darat, and *Hypnea pannosa* from Kusu Island) and Pulau Satumu where the *in situ* experiment was conducted (A). Gray shaded areas represent fringing reef areas. Photos of macroalgal species (B–E) and coral species (F–H) tested in this study.

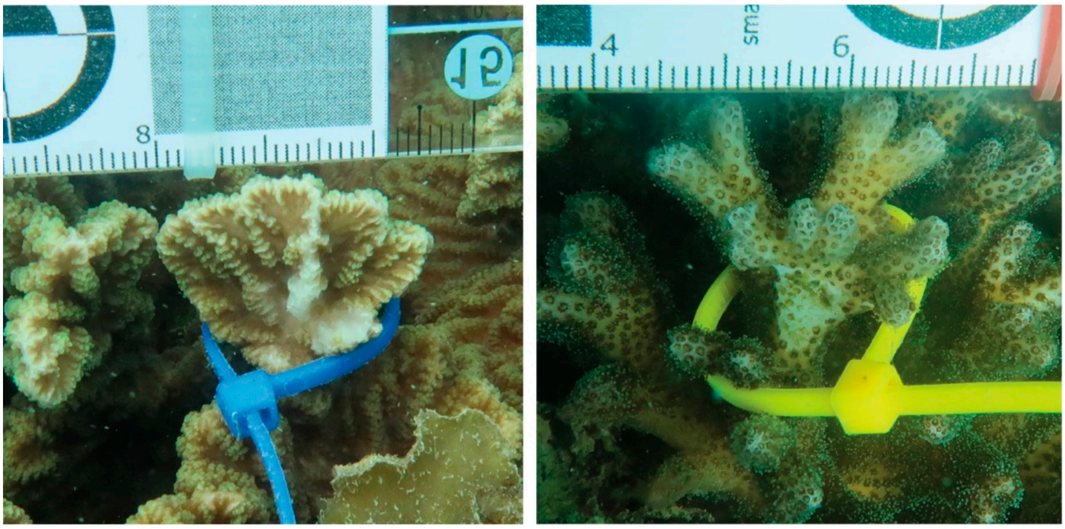

Figure S2. Photos of coral branches (left: *Merulina ampliata*; right: *Pocillopora acuta*) suffering from tissue bleaching due to exposure to macroalgal extracts (left: *Lobophora challengeriae*; right: *Hypnea pannosa*).

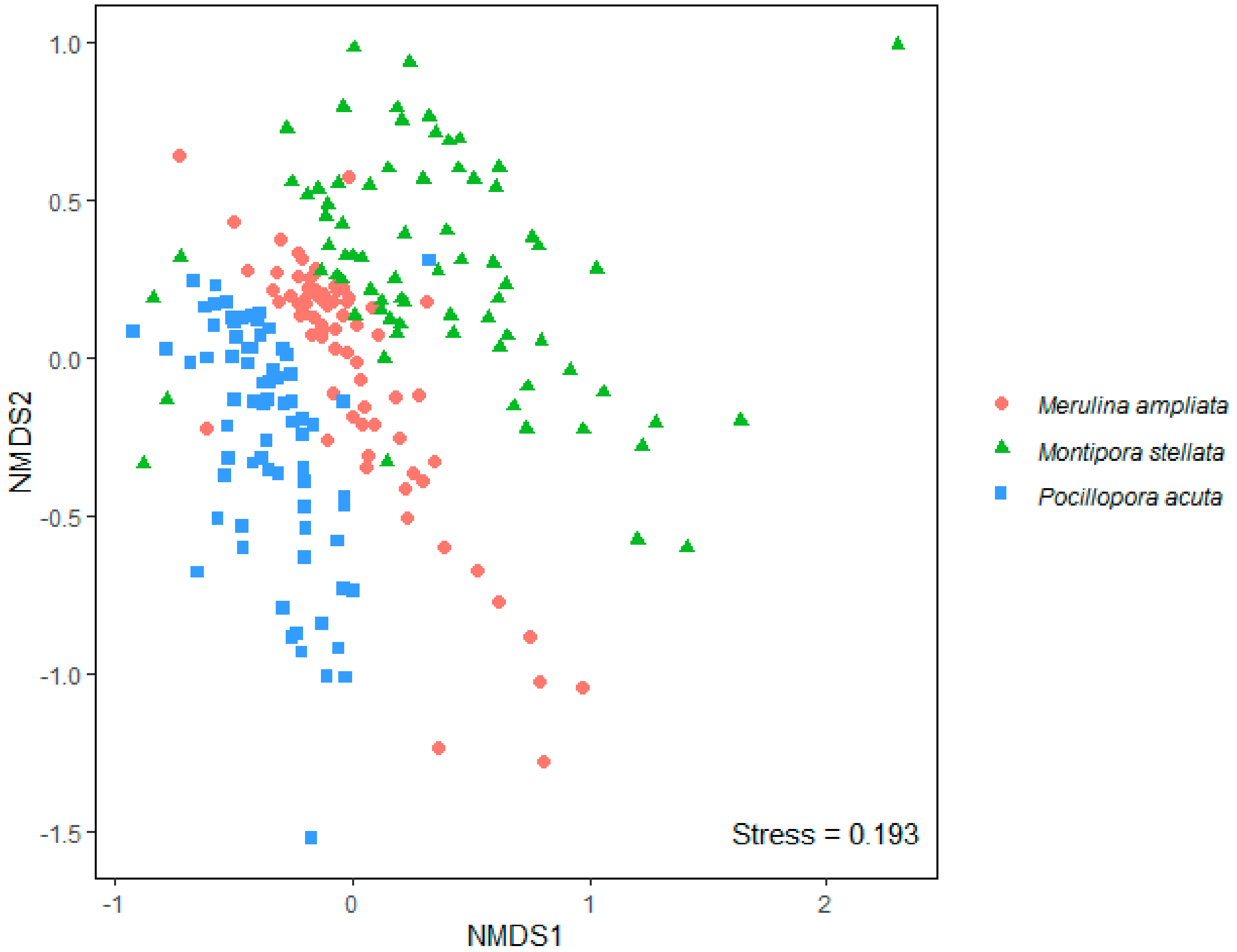

Figure S3. Non-metric multi-dimensional scaling (NMDS) plots based on Bray–Curtis distances comparing the coral microbiomes of the three coral species. The different colors indicate the three coral species used in this study.

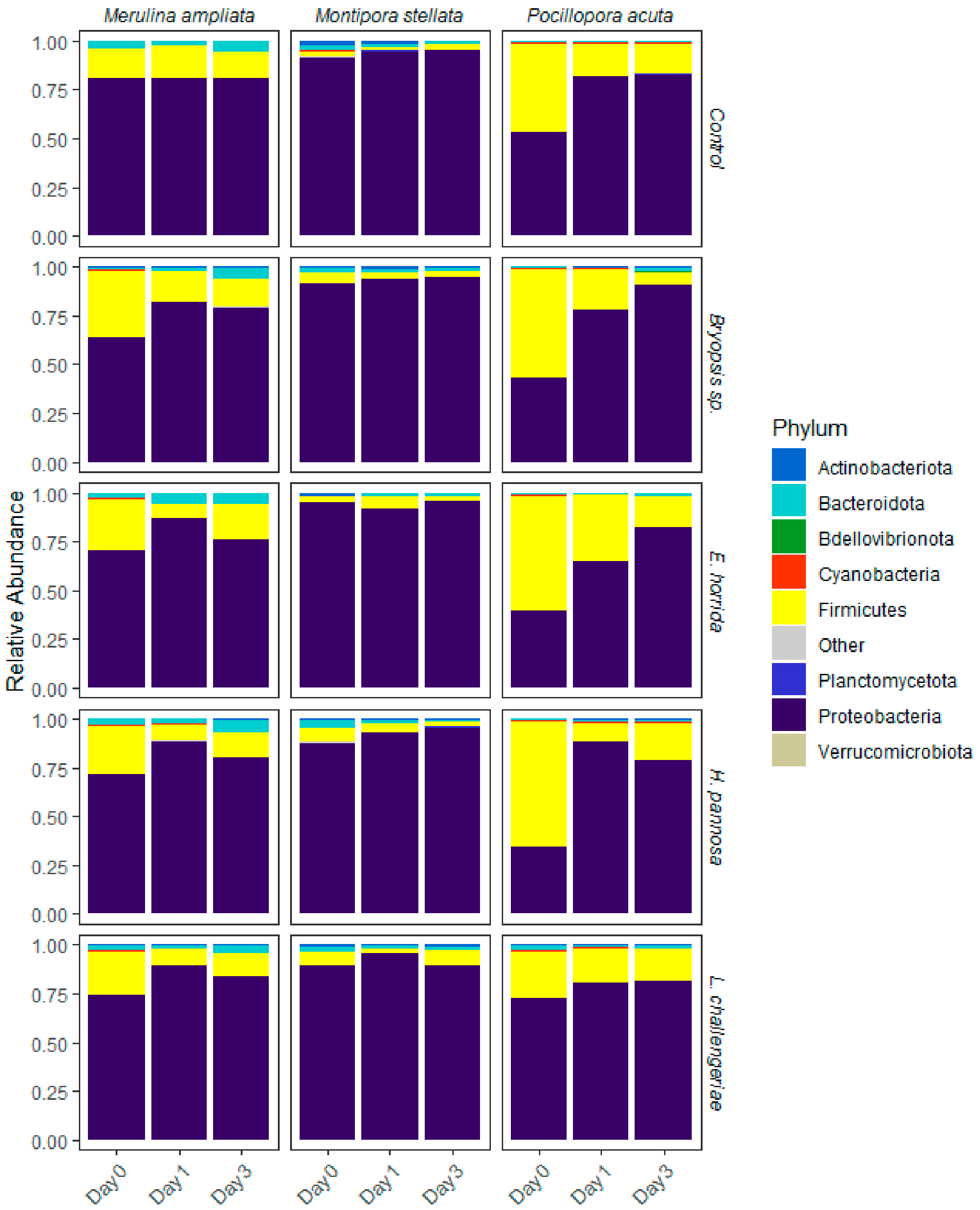

Figure S4: Relative abundances of the coral microbiomes (Phylum-level) of the three coral species that were in contact with macroalgal extracts across different time points.

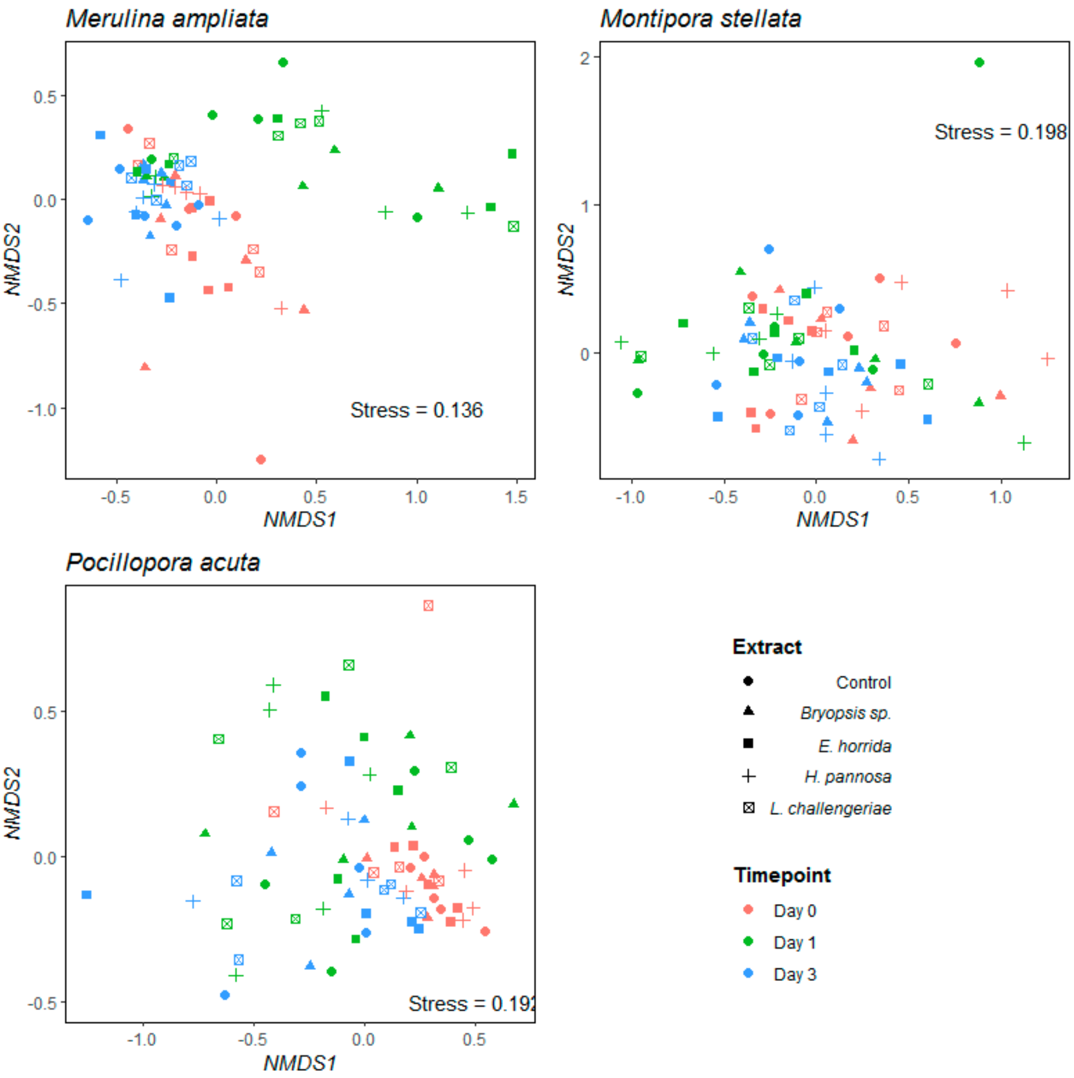

Figure S5: Non-metric multi-dimensional scaling (NMDS) plots showing the overall temporal shifts in the microbiomes of *M. ampliata*, *M. stellata* and *P. acuta*.

Table S1: Summary of the *DESeq2* analyses showing the ASV differences in *M. ampliata*, *M. stellata* and *P. acuta* microbiomes between the methanol control and macroalgal extracts at different time points.

ASV	Phylum	Lowest taxonomical rank	Coral species	Macroalgal extract	Time point	Log2 fold change
14	Proteobacteria	*Halomonas* sp.	*P. acuta*	*Bryopsis* sp.	Day 1	18.339
21	Proteobacteria	*Acinetobacter* sp.	*M. stellata*	*E. horrida*	Day 0	23.124
			P. acuta	*E. horrida*<br>*H. pannosa*<br>*L. challengeriae*	Day 0	-39.451<br>-38.538<br>-40.183
30	Proteobacteria	Family Rhodobacteraceae	*P. acuta*	*Bryopsis* sp.<br>*H. pannosa*<br>*L. challengeriae*	Day 1	20.197<br>32.068<br>31.620
34	Proteobacteria	*Halomonas* sp.	*M. stellata*	*Bryopsis* sp.	Day 0	23.689
41	Proteobacteria	*Sphingobium* sp.	*M. stellata*	*Bryopsis* sp.<br>*E. horrida*<br>*H. pannosa*<br>*L. challengeriae*	Day 3	-21.305<br>-22.020<br>-20.283<br>-21.585
46	Proteobacteria	*Comamonas* sp.	*M. stellata*	*E. horrida*	Day 0	23.439
47	Proteobacteria	*Erythrobacter sp*	*M. ampliata*	*Bryopsis* sp.<br>*H. pannosa*	Day 1	23.098<br>39.762
			M. stellata	*E. horrida*	Day 1	23.589
56	Firmicutes	*Exiguobacterium* sp.	*M. stellata*	*E. horrida*	Day 1	22.858
58	Proteobacteria	*Pseudomonas* sp.	*P. acuta*	*Bryopsis* sp.	Day 1	19.354
			M. ampliata	*Bryopsis* sp.<br>*E. horrida*<br>*H. pannosa*<br>*L. challengeriae*	Day 3	-22.438<br>-22.765<br>-21.990<br>-21.343
66	Proteobacteria	Family Rhodobacteraceae	*M. stellata*	*Bryopsis* sp.<br>*E. horrida*<br>*L. challengeriae*	Day 0	25.534<br>28.839<br>29.350
67	Firmicutes	Family Bacillaceae	*P. acuta*	*E. horrida*	Day 1	20.210
69	Firmicutes	*Planococcus* sp.	*M. stellata*	*L. challengeriae*	Day 0	22.821
			M. ampliata	*Bryopsis* sp.	Day 1	21.47

				L. challengeriae	Day 3	22.196
71	Proteobacteria	*Sphingomonas* sp.	*M. stellata*	*E. horrida*	Day 0	21.090
			M. ampliata	*Bryopsis* sp.<br>*E. horrida*<br>*L. challengeriae*	Day 1	-22.11<br>-21.651<br>-21.713
72	Proteobacteria	*Paracoccus* sp.	*M. ampliata*	*Bryopsis* sp.	Day 0	-21.58
75	Proteobacteria	*Paracoccus oceanense*		*H. pannosa*	Day 3	19.746
77	Bacteroidota	*Flavobacterium* sp.	*M. stellata*	*H. pannosa*	Day 3	25.186
78	Proteobacteria	*Stenotrophomonas* sp.	*P. acuta*	*Bryopsis* sp.	Day 1	17.461
82	Proteobacteria	*Massilia* sp.	*M. stellata*	*L. challengeriae*	Day 1	22.905
83	Proteobacteria	*Alteromonas australica*	*M. ampliata*	*L. challengeriae*	Day 3	23.977
			M. stellata	*H. pannosa*<br>*L. challengeriae*	Day 3	-27.204<br>-28.261
86	Proteobacteria	*Sphingomonas koreensis*	*M. stellata*	*E. horrida*	Day 0	19.963
89	Proteobacteria	*Paracoccus* sp.	*M. stellata*	*Bryopsis* sp.<br>*E. horrida*<br>*H. pannosa*	Day 1	-22.389<br>-22.785<br>-21.970
				E. horrida<br>*H. pannosa*	Day 3	24.664<br>24.393
92	Proteobacteria	*Acinetobacter* sp.	*M. stellata*	*Bryopsis* sp.	Day 0	23.056
				Bryopsis sp.<br>*E. horrida*<br>*H. pannosa*	Day 1	-22.336<br>-21.668<br>-20.631
94	Proteobacteria	*Yangia pacifica*	*M. stellata*	*Bryopsis* sp.<br>*E. horrida*<br>*H. pannosa*<br>*L. challengeriae*	Day 1	-22.664<br>-23.713<br>-23.135<br>-18.327
				H. pannosa	Day 3	23.497
95	Bacteroidota	*Mesoflavibacter* sp.	*M. stellata*	*L. challengeriae*	Day 0	25.332

96	Proteobacteria	*Ascidiaceihabitans* sp.	*M. stellata*	*Bryopsis* sp.<br>*E. horrida*<br>*H. pannosa*	Day 1	-22.336<br>-21.668<br>-20.631
			M. ampliata	*Bryopsis* sp.<br>*E. horrida*<br>*H. pannosa*	Day 3	-20.530<br>-23.142<br>-23.158
97	Proteobacteria	*Stenotrophomonas* sp.	*M. stellata*	*E. horrida*<br>*L. challengeriae*	Day 1	-31.690<br>-31.009
98	Proteobacteria	*Devosia* sp.	*P. acuta*	*Bryopsis* sp.	Day 1	21.204
99	Firmicutes	Family Bacillaceae	*M. stellata*	*Bryopsis* sp.<br>*E. horrida*<br>*H. pannosa*<br>*L. challengeriae*	Day 0	-21.439<br>-25.542<br>-20.077<br>-24.275
100	Proteobacteria	*Lysobacter spongiicola*	*M. stellata*	*H. pannosa*	Day 3	21.287
104	Bacteroidota	*Chryseobacterium* sp.	*M. stellata*	*E. horrida*	Day 0	19.142
				Bryopsis sp.<br>*E. horrida*<br>*H. pannosa*<br>*L. challengeriae*	Day 3	-19.374<br>-21.244<br>-21.655<br>-22.253
			M. ampliata	*E. horrida*	Day 3	23.683
111	Proteobacteria	*Paucibacter* sp.	*M. stellata*	*Bryopsis* sp.<br>*H. pannosa*<br>*L. challengeriae*	Day 0	-19.830<br>-22.682<br>-23.546
				L. challengeriae	Day 3	22.146
115	Firmicutes	*Lysinibacillus* sp.	*M. stellata*	*H. pannosa*	Day 0	22.073
				Bryopsis sp.<br>*E. horrida*<br>*H. pannosa*	Day 1	-22.632<br>-22.687<br>-19.732
118	Proteobacteria	*Brevundimonas* sp.	*M. stellata*	*Bryopsis* sp.<br>*E. horrida*<br>*H. pannosa*	Day 1	-19.271<br>-24.960<br>-22.395

				L. challengeriae		-23.187
			P. acuta	*Bryopsis* sp.	Day 1	-21.220
				E. horrida		-21.342
				H. pannosa		-22.201
				L. challengeriae		-20.983
125	Proteobacteria	*Sphingomonas dokdonensis*	*M. ampliata*	*E. horrida*	Day 1	23.593
				H. pannosa		23.832
132	Proteobacteria	*Acinetobacter schindleri*	*P. acuta*	*Bryopsis* sp.	Day 1	21.956
135	Bacteroidota	*Pontibacter* sp.	*M. stellata*	*E. horrida*	Day 0	22.323
137	Proteobacteria	*Ruegeria* sp.	*P. acuta*	*E. horrida*	Day 1	21.855
			M. ampliata	*E. horrida*	Day 1	-31.352
				L. challengeriae		-27.144
			M. stellata	*Bryopsis* sp.	Day 1	-21.407
				E. horrida		-22.461
				H. pannosa		-20.689
				L. challengeriae		-19.325
141	Bacteroidota	*Chryseobacterium montanum*	*M. stellata*	*Bryopsis* sp.	Day 0	18.593
142	Proteobacteria	*Allorhizobium-Neorhizobium-Pararhizobium-Rhizobium* sp.	*P. acuta*	*Bryopsis* sp.	Day 1	18.966
				E. horrida		39.637
143	Proteobacteria	*Pseudomonas* sp.	*M. stellata*	*H. pannosa*	Day 3	23.028
145	Proteobacteria	*Novosphingobium* sp.	*M. stellata*	*Bryopsis* sp.	Day 0	-21.480
				E. horrida		-19.107
				H. pannosa		-22.649
				L. challengeriae		-21.657
				H. pannosa	Day 1	20.247
				Bryopsis sp.	Day 3	-21.288
				E. horrida		-21.515
				H. pannosa		-20.496
				L. challengeriae		-21.872
146	Bdellovibrionota	*Peredibacter* sp.	*P. acuta*	*Bryopsis* sp.	Day 1	17.996

147	Proteobacteria	*Psychrobacter* sp.	*M. ampliata*	*Bryopsis sp*<br>*E. horrida*	Day 1	-23.37<br>-22.636
151	Firmicutes	*Staphylococcus* sp.	*M. stellata*	*Bryopsis* sp.<br>*E. horrida*<br>*L. challengeriae*	Day 0	-20.395<br>-23.154<br>-22.008
			P. acuta	*E. horrida*<br>*L. challengeriae*	Day 3	-29.577<br>-30.098
152	Proteobacteria	*Paracoccus* sp.	*M. stellata*	*Bryopsis* sp.	Day 3	18.487
157	Proteobacteria	Family Vibrionaceae	*P. acuta*	*Bryopsis* sp.<br>*E. horrida*<br>*L. challengeriae*	Day 1	-21.356<br>-17.312<br>-21.449
160	Bacteroidota	*Olleya* sp.	*M. ampliata*	*Bryopsis* sp.<br>*E. horrida*<br>*H. pannosa*	Day 3	-20.530<br>-23.142<br>-23.158
163	Proteobacteria	*Alteromonas* sp.	*M. ampliata*	*E. horrida*	Day 0	22.22
165	Proteobacteria	Family Rhodobacteraceae	*M. stellata*	*H. pannosa*	Day 0	21.469
				E. horrida<br>*L. challengeriae*	Day 3	-29.107<br>-34.362
172	Proteobacteria	*Aureimonas altamirensis*	*M. stellata*	*H. pannosa*	Day 3	23.555
177	Firmicutes	*Family Thermoactinomycetaceae*	*P. acuta*	*Bryopsis* sp.<br>*H. pannosa*<br>*L. challengeriae*	Day 1	-21.349<br>-19.479<br>-17.865
181	Proteobacteria	*Phenylobacterium* sp.	*M. stellata*	*E. horrida*	Day 3	21.121
182	Proteobacteria	*Roseomonas* sp.	*M. stellata*	*H. pannosa*	Day 1	19.267
183	Firmicutes	*Romboutsia sedimentorum*	*M. stellata*	*H. pannosa*<br>*L. challengeriae*	Day 1	19.191<br>20.561
			M. ampliata	*Bryopsis* sp.<br>*E. horrida*<br>*H. pannosa*<br>*L. challengeriae*	Day 1	-19.27<br>-20.655<br>-20.920<br>-22.221

185	Proteobacteria	*Comamonas* sp.	*M. stellata*	*L. challengeriae*	Day 3	23.254
188	Bacteroidota	Family Flavobacteriaceae	*M. stellata*	*Bryopsis* sp.<br>*E. horrida*<br>*L. challengeriae*	Day 0	21.938<br>29.271<br>29.793
191	Proteobacteria	*Brevundimonas* sp.	*M. stellata*	*H. pannosa*<br>*L. challengeriae*	Day 1	26.275<br>27.173
			M. ampliata	*Bryopsis* sp.<br>*L. challengeriae*	Day 3	20.873<br>33.538
192	Proteobacteria	Family Pseudomonadaceae	*M. stellata*	*Bryopsis* sp.<br>*E. horrida*<br>*H. pannosa*<br>*L. challengeriae*	Day 3	-20.957<br>-21.676<br>-18.365<br>-21.529
195	Proteobacteria	*Limimaricola* sp.	*M. stellata*	*Bryopsis* sp.<br>*E. horrida*<br>*H. pannosa*<br>*L. challengeriae*	Day 0	-23.287<br>-22.512<br>-20.930<br>-22.374
			P. acuta	*E. horrida*<br>*H. pannosa*	Day 1	20.191<br>20.670
196	Bacteroidota	*Vitellibacter* sp.	*M. stellata*	*Bryopsis* sp.<br>*H. pannosa*<br>*L. challengeriae*	Day 1	-22.993<br>-18.061<br>-17.972
				Bryopsis sp.<br>*E. horrida*<br>*L. challengeriae*	Day 3	-19.057<br>-19.773<br>-22.995
202	Verrucomicrobiota	Family Simkaniaceae	*M. ampliata*	*Bryopsis* sp.	Day 0	17.20
214	Proteobacteria	*Sphingomonas zeicaulis*	*P. acuta*	*E. horrida*	Day 1	21.172
218	Actinobacteriota	*Leucobacter* sp.	*M. stellata*	*H. pannosa*<br>*L. challengeriae*	Day 3	-26.742<br>-31.626
229	Firmicutes	*Epulopiscium* sp.	*P. acuta*	*Bryopsis* sp.	Day 1	18.869
230	Proteobacteria	Family Micavibrionaceae	*M. stellata*	*H. pannosa*	Day 1	22.884
233	Proteobacteria	*60(NOR5)_clade* sp.	*P. acuta*	*Bryopsis* sp.	Day 1	17.887

244	Bacteroidota	*Sphingobacterium* sp.	*P. acuta*	*Bryopsis* sp.<br>*E. horrida*<br>*H. pannosa*<br>*L. challengeriae*	Day 1	-21.961<br>-18.147<br>-20.589<br>-20.502
247	Firmicutes	Family Bacillaceae	*M. stellata*	*E. horrida*<br>*H. pannosa*<br>*L. challengeriae*	Day 3	-36.148<br>-39.382<br>-37.816
248	Actinobacteriota	*Brachybacterium* sp.	*M. stellata*	*Bryopsis* sp.<br>*E. horrida*<br>*H. pannosa*<br>*L. challengeriae*	Day 3	-16.780<br>-20.138<br>-24.651<br>-20.928
250	Bacteroidota	*Rubrivirga* sp.	*M. stellata*	*H. pannosa*	Day 0	21.953
				E. horrida<br>*L. challengeriae*	Day 3	-24.918<br>-28.760
251	Proteobacteria	*Candidatus_Endoecteinascidia* sp.	*M. ampliata*	*Bryopsis* sp.	Day 3	21.828
258	Bacteroidota	*Maribacter dokdonensis*	*M. stellata*	*Bryopsis* sp.<br>*E. horrida*<br>*L. challengeriae*	Day 0	20.194<br>29.537<br>30.404
262	Proteobacteria	*Altererythrobacter* sp.	*M. stellata*	*Bryopsis* sp.<br>*E. horrida*	Day 0	20.586<br>40.362
263	Proteobacteria	*Hyphomonas* sp.	*M. stellata*	*Bryopsis* sp.<br>*E. horrida*	Day 0	20.687<br>34.702
264	Proteobacteria	*Filomicrobium* sp.	*P. acuta*	*E. horrida*	Day 1	18.617
269	Proteobacteria	*Acinetobacter* sp.	*M. stellata*	*Bryopsis* sp.<br>*H. pannosa*	Day 0	-23.529<br>-19.418
276	Proteobacteria	*Alteromonas* sp.	*M. stellata*	*Bryopsis* sp.<br>*E. horrida*<br>*L. challengeriae*	Day 0	19.948<br>30.634<br>31.877
278	Firmicutes	Family Bacillaceae	*M. stellata*	*E. horrida*<br>*H. pannosa*<br>*L. challengeriae*	Day 3	-40.529<br>-41.267<br>-39.138

282	Proteobacteria	*Acinetobacter* sp.	*M. ampliata*	*E. horrida*<br>*H. pannosa*<br>*L. challengeriae*	Day 1	-40.199<br>-38.807<br>-40.316
285	Bdellovibrionota	*Peredibacter* sp.	*P. acuta*	*Bryopsis* sp.<br>*E. horrida*	Day 1	18.299<br>39.367
288	Proteobacteria	*Brevundimonas diminuta*	*P. acuta*	*L. challengeriae*	Day 1	24.718
320	Proteobacteria	*Methyloceanibacter* sp.	*P. acuta*	*E. horrida*	Day 1	19.308
326	Proteobacteria	*Enhydrobacter* sp.	*P. acuta*	*H. pannosa*<br>*L. challengeriae*	Day 1	-29.108<br>-27.749
333	Bacteroidota	*Aquibacter* sp.	*M. stellata*	*Bryopsis* sp.<br>*E. horrida*<br>*L. challengeriae*	Day 0	20.636<br>32.273<br>33.400
343	Proteobacteria	*Filomicrobium* sp.	*P. acuta*	*E. horrida*<br>*H. pannosa*	Day 3	-30.468<br>-29.110
401	Bacteroidota	*Rhabdobacter* sp.	*M. stellata*	*E. horrida*	Day 0	21.213
409	Proteobacteria	*Roseovarius* sp.	*M. stellata*	*E. horrida*<br>*L. challengeriae*	Day 0	-29.561<br>-31.236
415	Proteobacteria	*Aureimonas* sp.	*M. ampliata*	*Bryopsis* sp.<br>*H. pannosa*	Day 1	-21.48<br>-19.501
450	Verrucomicrobiota	*Prosthecobacter* sp.	*P. acuta*	*E. horrida*	Day 3	39.499
501	Firmicutes	*Epulopiscium* sp.	*M. stellata*	*E. horrida*<br>*H. pannosa*<br>*L. challengeriae*	Day 1	-34.557<br>-38.450<br>-37.131
